# Telerehabilitation in Community Stroke Services: Mixed Methods Evaluation of Current Practice and Lessons for Sustained Use

**DOI:** 10.2196/87741

**Published:** 2026-06-11

**Authors:** Elizabeth Chandler, Charlotte Dorer, Valerie M Pomeroy, Nicola J Hancock

**Affiliations:** 1 School of Health Sciences, Faculty of Medicine and Health Sciences, University of East Anglia, The Queen's Building, Norwich Research Park, Norwich, England, NR4 7TJ, United Kingdom, 44 0 1603 593316; 2 NHS (National Health Service) England–East of England, Cambridge, United Kingdom; 3 Department of Clinical Neurosciences, University of Cambridge; NIHR (National Institute for Health and Care Research) HealthTech Research Centre in Brain Injury, Cambridge, United Kingdom

**Keywords:** stroke, telerehabilitation, stroke rehabilitation, community services, service innovation

## Abstract

**Background:**

The delivery of specialist stroke rehabilitation is undergoing a significant transformation, with telerehabilitation increasingly integrated into clinical practice and supported by guidelines and policy. There is a need for the pragmatic evaluation of telerehabilitation in service, which includes insights from clinical teams and people with stroke. This evaluation sought to address that need in the context of community stroke services in the East of England.

**Objective:**

Our evaluation addressed two over-arching aims: (1) to investigate and map contemporary models and experiences of telerehabilitation delivery in community stroke services, examining how it is currently used and perceived by both health care providers and service-users; and (2) to identify practical lessons and enabling factors that support the sustained integration of telerehabilitation into routine community stroke services.

**Methods:**

This study is a two-phase exploratory sequential mixed methods evaluation. Phase one involved discussion groups with stakeholders already using telerehabilitation to explore experiences, attitudes, influences, and behaviors associated with its use. Findings from phase one directly informed phase two by the development of a conceptual framework and in shaping the content of an online survey for clinicians and people with lived experience of stroke. Quantitative and qualitative data were subsequently integrated through triangulation during analysis, interpretation, and reporting stages. Data from the discussion groups were analyzed using a recognized framework for reflexive thematic analysis within a contextualist approach. Descriptive statistics were used to summarize quantitative survey responses.

**Results:**

A total of 20 people attended the discussion groups (n=4 groups total). Further, 96 people responded to the survey. Three themes underpinning successful use of telerehabilitation in this context were derived from triangulation across our data sources: (1) consideration of risks and benefits, with fewer than half of staff viewing telerehabilitation as equivalent in safety (22/49, 45%) or effectiveness (18/49, 37%), but most reporting that they could build rapport remotely (34/49, 69%); (2) the importance of individualized care approaches, where most clinicians reported confidence in identifying which service-users would benefit from telerehabilitation (42/49, 85%), although only 20% (10/49) offered it routinely; and (3) the need for staff support, with up to 34% of staff reporting no training in how to assess suitability for telerehabilitation. Key insights included the potential for telerehabilitation to increase efficiency and address service pressures, the importance of addressing digital exclusion, the value of individualized approaches, and the need for timely and tailored staff training.

**Conclusions:**

Our pragmatic, in-service evaluation demonstrates that telerehabilitation works best not as a replacement for in-person care, but as part of a responsive, blended approach grounded in individual need. These findings highlight that, with appropriate clinician training and flexibility in delivery, telerehabilitation can meet the needs of individuals through personalized approaches while supporting service responsiveness in pressurized clinical environments.

## Introduction

Telerehabilitation is defined as the delivery of rehabilitation consultations, assessments, and therapies remotely using information and communication technologies [1]. It can involve real-time remote consultation, as well as the sharing of information such as therapy programs and monitoring performance at other times. The possibilities for telerehabilitation to support provision of stroke rehabilitation were explored in a 2020 systematic review and meta-analysis of 22 studies published between 2004 and 2019: comparisons of a small number of studies found that telerehabilitation might be as effective as in-person care at least for activities of daily living (n=2 studies, moderate-quality evidence) and upper limb function (n=3 studies, low-quality evidence) [2]. Telerehabilitation has been proposed as a possible solution to some identified challenges of community rehabilitation, such as remote geographical locations [3].

The use of telerehabilitation was rapidly accelerated by the COVID-19 pandemic, with clinical stroke teams adapting to digital solutions for rehabilitation and care delivery, to continue the provision of some rehabilitation when face-to-face contact was minimal. Following this rapid adoption, telerehabilitation is now an increasing part of specialist stroke rehabilitation delivery and is supported as such in guidelines and policy [4,5]. Two of the UK government’s 10-year health plan “big shifts”—policy drivers initiated after the COVID-19 pandemic—speak directly to increased use of delivery beyond hospital settings: “analogue to digital” and “hospital to community care.” Fundamental to those shifts is digital transformation to enhance patient care through the use of technology [6]. As a result, the landscape of stroke rehabilitation is changing.

Despite such policy-driven support for telerehabilitation, it is recommended that further pragmatic evaluation of its use, including understanding patient experiences and those of clinical teams, is carried out to support its implementation as a mode of delivery for stroke rehabilitation [7]. Further evaluation, monitoring, and engagement with service users is considered critical to digital service improvement [5]. Following a period of enforced change, there is a need now to strengthen the evidence and understanding in parallel to the service delivery change [7,8]. While safety and feasibility of specific teledelivered stroke programs have been explored, for example, for intense upper limb rehabilitation [9] and for speech and language therapy [10], understanding of how to operationalize telerehabilitation in community stroke rehabilitation and sustain its use is limited [11]. A deeper exploration of the foundational components of successful use of telerehabilitation is required.

To meet the challenge of evaluation alongside existing service delivery, the service evaluation reported here was commissioned by NHS (National Health Service) East of England as part of the UK-wide Stroke Quality Improvement for Rehabilitation initiatives to support the delivery of the UK Integrated Community Stroke Service [5]. The East of England provided a particularly rich setting for the work, due to community health provision across a mix of rural, suburban, and city-based populations.

The overarching aims of the mixed methods evaluation presented here were to (1) investigate and map contemporary models and experiences of telerehabilitation delivery in community stroke services, examining how it is currently used and perceived by both health care providers and service-users; and (2) identify practical lessons and enabling factors that support the sustained integration of telerehabilitation into routine community stroke services.

Specific evaluation questions were the following: (1) In what ways is telerehabilitation currently delivered in community stroke services, and how do experiences vary between providers and service-users? (2) What are the key components of successful use of telerehabilitation in community stroke rehabilitation practice?

## Methods

### Study Design

An exploratory sequential mixed methods evaluation was conducted between April 2023 and March 2024. A mixed methods evaluation was used due to the known complexity of pragmatic health services evaluation, enabling deep insights unavailable to the evaluation team from either quantitative or qualitative data alone [12]. The current study is reported with attention to the GRAMMS (Good Reporting of a Mixed Methods Study) reporting guidelines for mixed methods health service research [13] (Multimedia Appendix 1).

To address study aims, the evaluation comprised two phases: regarding phase one, discussion groups with clinicians working in community stroke services, the voluntary sector, and members of an established stroke-specific patient and public involvement (PPI) group; and regarding phase two, conceptual framework development and an online survey for clinicians and people with lived experience of stroke.

Using the sequential exploratory design, findings from phase one directly informed and were connected to phase two by shaping the conceptual framework and survey content. Quantitative and qualitative data were subsequently integrated through triangulation during analysis, interpretation, and reporting stages.

### Inclusion and Exclusion Criteria

Eligibility criteria were necessarily broad to reflect the range of perspectives across community stroke services. Clinicians working in stroke services in the East of England and PPI members with experience of stroke rehabilitation in the region were eligible to take part. Participation in phase one did not preclude participation in phase two. All were invited to take part in both phases of the evaluation, with the option to take part in phase two regardless of participation in phase one.

For phase one, purposeful sampling was used to recruit clinicians with experience of delivering telerehabilitation, to inform the development of the conceptual framework and survey.

Responses from participants working only in acute services were not included in the analysis.

### Recruitment

Recruitment for phase one was conducted via clinical and stroke-specific PPI meetings. At these meetings, the evaluation team presented this study and its aims and invited attendees to participate. Individuals interested in taking part were asked to contact the evaluation team by email to request a participant information sheet with full details of the evaluation and consent procedure (phase one). All participants had the opportunity to ask questions, and written informed consent was obtained.

To maximize accessibility for PPI members, this study was presented at two meetings held at different times of the day.

Fifteen community stroke teams were invited to the clinical meeting; services represented were located within two eastern integrated stroke delivery networks in England.

Phase two differed from phase one in that participation involved the completion of an anonymous survey online rather than enrollment in discussion groups. The survey was accessible via an online link or a QR code. Participation was voluntary, and an introductory page outlined the purpose of this study, data handling practices, and participants’ right to withdraw at any time before submission. Consent was implied through survey completion and submission. Participation was advertised at clinical and PPI meetings, as in phase one, but at a later date. To extend the reach across the region, the survey was also promoted at the East of England Stroke Forum and shared via two stroke charities.

### Researcher Context and Reflexivity

Data from the discussion groups were analyzed using reflexive thematic analysis within a contextualist approach, enabling flexibility of combining multiple sources of data such as people’s experiences and practices [14,15]. This approach highlights the researcher’s active role in knowledge production. Codes are understood to represent the researcher’s interpretations of patterns of meaning across the dataset. Understanding evaluators’ backgrounds in relation to the phenomenon at hand is an important aspect of conducting reflexive qualitative evaluation. Here, the lead author (EC) is a chartered physiotherapist and has worked in NHS community and stroke rehabilitation services. EC conducted all the discussion groups. The principal evaluator (NJH) is a chartered physiotherapist, researcher, and innovation lead working in Higher Education, with extensive experience in NHS stroke rehabilitation service delivery, evaluation, and research. These professional perspectives informed the interpretation of the data, and ongoing reflexive discussion was used to consider how our assumptions and experiences influenced the theme development. NJH was present for 2 of 4 discussion groups. Participants were aware of the evaluators’ background when taking part in the discussion groups.

### Data Collection

#### Phase One

Discussion groups were used to explore the use and experiences of telerehabilitation and to identify key attitudes, influences, and behaviors associated with its use. A topic guide was developed a priori (Multimedia Appendix 2: Discussion groups topic guide).

At the start of each discussion group, participants were informed that the conversation would be recorded, reminded of their right to withdraw, and assured that their identities would be pseudo-anonymized.

Four groups were held, facilitated by EC. A choice of online or in-person discussion was offered, resulting in three via videoconferencing (Microsoft Teams [Microsoft Corp]) and one in-person. PPI members and clinical staff attended together to make use of group dynamics, encourage rich discussion, and explore complex issues from both viewpoints. The facilitator used inclusive techniques to support all voices being heard (eg, inviting responses from all participants and managing dominant speakers). Groups were recorded and then transcribed verbatim by an independent third party. Transcriptions were reviewed by EC for accuracy.

Discussion groups were conducted using a pragmatic, time-limited approach consistent with an in-service evaluation. Although formal data saturation was not predefined, few new points of discussion arose in the final group, suggesting adequate coverage of the key perspectives.

#### Phase Two

**Figure 1.**
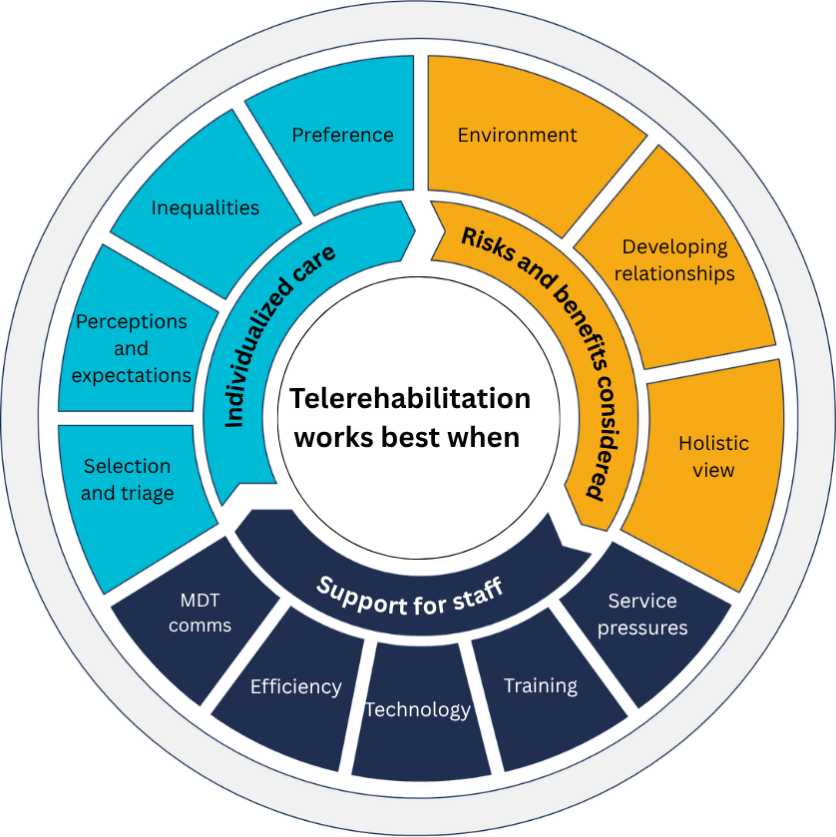
Conceptual diagram illustrating key themes underpinning successful use of telerehabilitation in community stroke services, developed from qualitative interviews with survivors of stroke and rehabilitation clinicians in a mixed methods study conducted in community neurorehabilitation services in the east of England, UK, 2023-2024. MDT comms: multidisciplinary team communication.

Findings from phase one were combined with insights from published literature and clinical guidelines to inform the development of a conceptual framework (Figure 1) and guide the design and content of an online survey. This framework ensured that all relevant topics and variables of interest were addressed, while also facilitating data triangulation by integrating multiple sources and perspectives. The survey aimed to evaluate the current use of telerehabilitation in community stroke services and to explore the beliefs and attitudes influencing its sustained use.

The survey was created using Microsoft Forms (Microsoft Corp) and piloted to test ease of use. It was designed to be mobile device-friendly and take no more than 20 minutes to complete. Filtering logic was used to customize questions based on each respondent’s role (clinicians providing stroke services, service users, and unpaid caregivers) and level of experience with telerehabilitation. Responses were anonymous, and no identifying information or IP addresses were collected.

The survey adopted a recommended range of data-gathering styles [16], including open- and closed-ended questions, ranking questions, and 4-point Likert scales with the options: strongly disagree, disagree, agree, and strongly agree.

It was accessible for eight weeks.

### Data Analysis

The reflexive thematic analysis was conducted by working iteratively through a six-phase process of data familiarization, generation of initial codes, generating themes, and reviewing, refining, and naming the themes [15]. According to Braun and Clarke [15], coding was conducted inductively, with codes generated from the data rather than a predefined framework.

Qualitative data were analyzed using an inductive coding approach, with codes generated directly from the data. Following coding, relevant published literature and clinical guidelines (including those from related areas of physiotherapy practice) were reviewed to check for potential gaps. This process did not lead to the identification of additional codes.

EC was responsible for all coding and generating themes. EC and NJH then worked collaboratively to reexamine and redefine themes, achieving richer interpretations of the meaning. Differences in interpretation were explored through discussion to deepen understanding and refine themes, consistent with a reflexive approach.

Quote identifiers in the results section include the discussion group number and the participant’s role (eg, DG1, OT for an occupational therapist in discussion group 1). Roles include: OT (occupational therapist), physiotherapist (physiotherapist), SLT (speech and language therapist), RA (rehabilitation assistant), SU (service-user with lived experience of stroke), and UC (unpaid caregiver).

Survey data were exported from Microsoft Forms into Microsoft Excel (Microsoft Corp) for analysis. Responses were screened for eligibility, and ineligible submissions (where rehabilitation or service experience did not take place in the East of England) were excluded. Missing data were reviewed; if a respondent left a nonessential question unanswered, their other responses were retained. No imputation methods were used to estimate missing values.

Where contradictory responses were identified, these were reviewed for internal consistency. In one case, a participant reported discontinuing telerehabilitation entirely but also provided detailed examples of its use. Their response was amended to reflect internally consistent use, in line with the examples given.

Descriptive statistics, including frequencies and percentages, were used to summarize demographic characteristics and closed-ended responses. Likert-scale items were summarized using the proportion of respondents selecting each response option.

For questions that allowed respondents to select multiple answers, percentages of respondents who selected each option as part of their responses were calculated.

The ranking data were analyzed by calculating weighted scores for each option based on frequency and rank position. For each response, items ranked in first position were assigned a score of 10, second position a score of 9, and so on down to a score of 1 for the lowest rank. Scores were then summed across responses to produce an overall score for each factor, with higher scores indicating greater perceived importance.

### Data Integration

Integration took place at both the design (building) and analysis (merging) stages. Findings from the phase one discussion groups were used to develop a conceptual framework, alongside relevant published literature, and this informed the design of the phase two questionnaire.

After data collection, findings from both phases were considered together. This allowed comparison of perspectives across the two datasets and helped identify areas of agreement and difference.

### Ethical Considerations

Ethical approval for this evaluation was granted by the University of East Anglia Faculty of Medicine and Health Sciences Research Ethics Service (ETH2223-1521).

All participants in the phase one discussion groups received a participant information sheet, had the opportunity to ask questions, and provided full informed written consent.

For phase two, the online survey was anonymous. An introductory section outlined the purpose of the evaluation, ethical approval, and data handling. Completion and submission of the survey were taken as implied consent.

Discussion group participants who traveled to the venue were entitled to reimbursement of their travel costs, but participants were not offered financial incentives to take part.

All data were securely stored on encrypted servers in compliance with regulatory requirements.

## Results

### Participants

#### Phase One: Discussion Groups

A total of 20 people attended across all discussion groups. They were occupational therapists (n=5), physiotherapists (n=3), speech and language therapists (n=4), rehabilitation assistants (n=4), members of the patient and public volunteer assurance group (n=2; both with lived experience of stroke but not telerehabilitation), and charity representatives from the Stroke Association and Different Strokes (n=2, one a survivor of stroke, also without telerehabilitation experience).

#### Phase Two: Online Survey

A total of 96 people responded to the survey. Four responses were excluded as the participants had not received stroke rehabilitation in the East of England, leaving 92 valid responses from multidisciplinary team (MDT) members (n=81), individuals who had experienced a stroke (n=10), and 1 unpaid caregiver.

Among the MDT respondents, 14 (17%) worked in acute stroke services, 38 (47%) in early supported discharge teams, and 29 (36%) in community stroke or community neurological services, including stroke rehabilitation. To meet the evaluation aims, only responses from clinical staff working in early supported discharge and community services are reported here (n=67).

Of the survivors of stroke, only one had received telerehabilitation services, while the unpaid caregiver had also experienced telerehabilitation.

To establish MDT participants’ experience with telerehabilitation as a baseline for understanding their perspectives and practices, a foundational question, “Have you ever used telerehabilitation?” was asked. A total of 49 (73.1%) of participants had used telerehabilitation (Tables1  2).

Unless otherwise stated, the findings relating to clinical staff reported are based on respondents who reported prior use of telerehabilitation (n=49), as survey filtering directed subsequent questions to this group. Responses to training-related questions, however, include all clinical participants regardless of telerehabilitation experience (n=67). Service user views are presented alongside clinician findings where available, but are based on small numbers (survivors of stroke n=10; caregiver n=1). Percentages are presented with underlying counts (n/N) to aid interpretation.

**Table 1.** Telerehabilitation use by profession among clinicians involved in community stroke rehabilitation services in the East of England, 2023-2024 (total clinician analytic sample, N=67).

Profession	Used telerehabilitation (n=49)	Never used telerehabilitation (n=18)	Total respondents (N=67)
Nonregistered staff	12	4	16
Occupational therapists	11	5	16
Speech and language therapists	11	0	11
Nurses	3	1	4
Psychology	6	1	7
Physiotherapists	4	5	9
Team leads	2	0	2
Dietitian	0	1	1
Therapist (unspecified)	0	1	1
Total	49	18	67

**Table 2.** Survey respondent groups (clinicians, survivors of stroke, and caregivers; total respondents N=92).

Respondent group	Values, n
MDT^a^ staff from community stroke services	29
MDT staff from early supported discharge	38
MDT staff from acute stroke services	14
Survivors of stroke	10
Unpaid caregivers	1
Total valid respondents	92

a MDT: multidisciplinary team.

### Evaluation Findings

#### Overview

A conceptual model was developed based on thematic analysis of phase one discussion group findings (Figure 1) and subsequently informed the design of the phase two survey. This analysis led to three derived themes. To enhance coherence, evaluation findings are here presented within these themes, integrating evidence from phase one (qualitative) and phase two (quantitative) data. Within each theme and subtheme, discussion group findings are presented first, followed by survey results, before being integrated through narrative and considered together.

It is important to note that some overlap exists between themes, reflecting the interconnected and nuanced nature of the factors influencing the use of telerehabilitation.

#### Theme One: Successful Use of Telerehabilitation—Risks and Benefits

##### Overview

This theme explores the importance of understanding both the potential and the limitations of telerehabilitation in community stroke services. Clinician reflections suggest that when delivered in isolation, telerehabilitation could increase risk, particularly in relation to safety, safeguarding, and clinical decision-making. However, when integrated within a broader service model, such as blended delivery following face-to-face contact, it is seen as a valuable adjunct to care.

As one participant described:

*On its own, well, I think it can expose risk, but if it’s used again as a blended approach after you’ve met face to face and you’ve seen the environment and you’ve established what support and what relationships there are around them, then you can move forward kind of knowing that you’re not missing anything.*

[DG2, SLT 1]

This balance between risk and benefit was echoed across the focus groups and shaped three interconnected subthemes: the environment, relationships, and working holistically.

##### The Environment

Concerns about missing key details in the home environment were consistently raised. Staff spoke of the limitations of video consultations in capturing the full context of a person’s living situation details as compared to face-to-face observations.

An occupational therapist reflected:

*The little things, like just the space around the toilet, you know, and there’s just things you don’t always pick up on that you can risk assess in the home environment sometimes.*

[DG3, OT1]

Survey responses (phase two) reflected divided views. Among staff who had used telerehabilitation, 53% (26/49) agreed that initial assessments should always be delivered face-to-face, while a similar proportion disagreed (23/49, 47%). Taken together with discussion group accounts, this suggests ongoing uncertainty about when telerehabilitation is appropriate and when in-person assessment is essential for safety and risk management.

##### Relationships

Participants reflected on how telerehabilitation alters the process of building therapeutic relationships. Many described a more limited sense of the individual and their social environment when interacting remotely.

As one clinician explained:

*The image, the impression that you get through the video or through the phone is very one-dimensional. So you only get the presentation of that person within that square and you don’t get those environmental clues. The relationships between the family or carers. So really those things that we pick up on the minute that we’re walking up to somebody’s house, we don’t get. The difference between face to face and sort of virtual options is that you don’t pick up on those other clues, those other things around the person.*

[DG2, SLT1]

Survey responses (phase two) reflected this complexity. A strong majority (34/49, 69%) agreed they could build rapport using telerehabilitation, although around 1 in 3 (15/49, 31%) still expressed doubt, suggesting that while feasible, clinical staff still experienced challenges in rapport-building remotely.

##### Working Holistically

For many staff, the ability to work holistically, seeing beyond the immediate clinical task, was perceived to be limited by remote delivery. In-person visits often revealed issues not directly related to the stroke-specific goal, enabling a more comprehensive and person-centered response.

One participant explained:

*I work with certain colleagues who … would really prefer not to do virtual because they don’t feel that they have the capacity to do that holistic approach in terms of reaching out beyond the actual stroke specific goal and beyond their professional boundaries in terms of you know what their quality of life is looking like and what kind of what’s the bigger picture? Basically, both in the immediate home environment, but also kind of their day-to-day?*

[DG1, PT1]

Survey findings (phase two) aligned with these concerns. Although 73% (36/49) of staff felt telerehabilitation helped them support the long-term needs of service-users, fewer felt it was as effective (18/49, 37%) or as safe (22/49, 45%) as face-to-face rehabilitation. This aligns with service-user perspectives (phase two), fewer than half of the survivors of stroke and unpaid caregivers surveyed (4/11, 36%) agreed that telerehabilitation was of equal quality to face-to-face therapy, while the majority (7/11, 63%) disagreed or strongly disagreed. Although the survey did not explore their reasoning in depth, these responses suggest that some individuals may have experienced limitations or perceived telerehabilitation as less personal or effective. Coupled with findings from clinicians that recognized the benefits of telerehabilitation, but also its limitations when striving for comprehensive care, these connected findings emphasize the importance of aligning telerehabilitation with individual preferences and ensuring it is not positioned as a universal substitute for in-person care. This is explored further in theme two.

In summary, theme one highlights the complex interplay of risks and benefits when using telerehabilitation in community stroke services. While many staff valued its flexibility and potential to reduce service burden, they also emphasized the need for careful implementation, particularly in relation to understanding the home environment, building relationships, and delivering holistic care. Juxtaposing the perspectives of survivors of stroke and caregivers further underscored that perceived quality and therapeutic connection may be diminished when services are delivered remotely, even when practical needs are met.

Survey items related to this theme are summarized in Multimedia Appendix 3: Survey items theme 1.

### Theme 2: Successful Use of Telerehabilitation—Individualized Care

#### Overview

This theme explores how telerehabilitation was shaped by individual needs, preferences, and circumstances. Staff and service users alike described a complex mix of practical, emotional, and contextual factors that influenced whether it was perceived as acceptable or beneficial. The legacy of the COVID-19 pandemic remained a notable influence on service delivery and personal choice. One clinician reflected:

*They’re still scared of Covid, so they still want their therapy, but they are like, “I don’t want people in my home.” Still scared and if they’ve got like someone in their home that’s unwell as well I think video works well. At least having a nice balance between the two so you are going in once a week, face-to-face but then having like the video calls I think sometimes just eases that anxiety.*

[DG4, RA2]

Participants emphasized that there was no single “type” of service user for whom telerehabilitation would always be appropriate or inappropriate. Instead, they described the importance of tailoring decisions individually. Interrogation of this theme identified four interrelated subthemes: selection and triage, perceptions and expectations, inequalities, and preferences.

#### Selection and Triage

Across all staff discussion groups, participants described how they triaged service users to determine suitability for telerehabilitation. Clinical factors such as cognitive impairment, visual or hearing loss, and severe communication difficulties were frequently cited as “red flags” for which telerehabilitation was likely to be inappropriate.

*It’s mainly, receptive or cognitive. Or they got visual or hearing [loss]. Those kind of red flags*.

[DG3, SLT 1]

Practical issues also influenced decision-making. For example, where service users lived at a considerable distance from the team, video appointments were sometimes used to supplement face-to-face visits. This approach was seen to increase capacity, particularly for disciplines such as speech and language therapy.

*If someone lives an hour’s drive, you’ve got to work that in your day, where on the phone you haven’t got to.*

[DG4 RA5]

Telerehabilitation was also used for brief, focused reviews, particularly where the service user’s needs were straightforward.

*Some people prefer to just have a quick review… They can just sort of sign in to the virtual waiting room to discuss their programme*.

[DG2, PT1]

In the survey findings (phase two), over one-third (18/49, 37%) of staff said they offered telerehabilitation selectively, based on whether they believed the person might like it. Only 20% (10/49) said they routinely offered it to all service users. When asked to rank suitability criteria, staff prioritized access to IT equipment, service user preference, and ability to engage in an online conversation above factors such as age, geography, or presence of a caregiver.

#### Perceptions and Expectations

Clinicians described a shift in how telerehabilitation was perceived, both by staff and service users, since the early days of the pandemic. While some still expressed concern that service users might see telerehabilitation as inferior, others noted positive changes in how expectations were managed and how individuals had adapted.

*I think a lot of our patients do have certain expectations of what they think therapy is gonna be… I still think they prefer face to face… Maybe they do feel they’re not getting a good deal*.

[DG3, SLT1]

However, others gave examples of how telerehabilitation could support self-management and build confidence:

*They are writing down problems before the phone call because they get a little letter before… I think their self-management skills have got better. I think it has helped with their expectations and they’re actually quite pleased that they are self-managing a bit better...*

[DG3, OT1]

Survey responses (phase two) indicated that 85% (42/49) of staff felt confident identifying who would benefit most from telerehabilitation, only 41% (20/49) believed that service users enjoyed it, and just 14% (7/49) thought it was considered equal in quality to face-to-face therapy. Among survivors of stroke and caregivers, 81% (9/11) said they would be comfortable using telerehabilitation, 100% (11/11) said they wanted to be involved in the decision to use it, and a slightly lower proportion (8/11, 73%) felt confident using technology, suggesting that willingness to engage with telerehabilitation may extend beyond self-confidence in the use of digital technology. Juxtaposition of the views of the clinicians and service users illustrates the importance of dialogue and shared understanding.

#### Inequalities

Concerns about inequality, particularly digital exclusion, were raised in every discussion group. Some participants reflected on assumptions about age and digital literacy, noting that these did not always hold true in practice:

*We have a cohort of our patients that are older. So there’s that perception that they wouldn’t be able to manage with Telerehab. And I think actually we were quite surprised how well that worked.*

[DG3, PT1]

*You assume a 35- or 40-year-old is going to be all right with the tech… but some people don’t [have Wi-Fi] and then you’ve got your 98-year-old and they’ve got their iMac*.

[DG4, RA5]

Others were more cautious, noting that older adults may prefer face-to-face contact to reduce isolation or maintain connection:

*It’s the contact, the physical contact. Well, especially for the older people… and that they’re not alone. As such, when it comes to the rehab support*.

[DG3, OT1]

Access to equipment was another recurring theme. Staff described how many service users lacked suitable devices or only had limited phone access.

*So many didn’t have any devices… a mobile phone, but it wasn’t a smartphone, and they’d only use it very occasionally*.

[DG1, SLT1]

**Figure 2.**
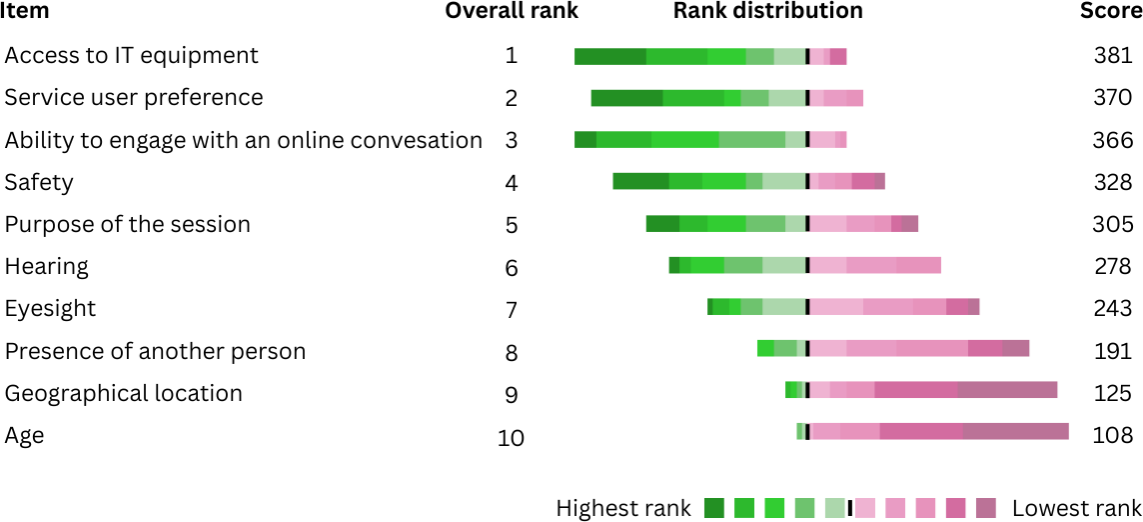
Diverging stacking bar-chart of clinical staffs’ ranking of 10 factors influencing telerehabilitation suitability decisions for survivors of stroke in community rehabilitation services, derived from quantitative survey data in the mixed methods survey of UK rehabilitation professionals, 2023-2024. Findings are from respondents reporting prior use of telerehabilitation (n=49). Factors are ordered by overall ranking score calculated from the frequency of assignment to each rank position across the 10-item ranking task, with higher ranks contributing greater weight.

These inequalities were also evident in the survey (phase two). Among the 10 factors ranked for telerehabilitation suitability, access to IT equipment was rated highest in importance, followed by service user preference, whereas age, geographical location, and presence of a caregiver were ranked lowest (Figure 2). Factors relating to safety, purpose, and communication occupied the midrange.

#### Preferences

Personal preference emerged as a key determinant of successful telerehabilitation. Staff spoke of making assumptions about who would or would not want remote appointments, often based on age or perceived technical ability. However, many recognized the need to move beyond assumptions and to ask service users directly.

*We probably sort of use a bit of bias and maybe in the patients that we think you know would prefer to do video consultations and maybe ask them*.

[DG3, SLT2]

Several clinicians described how shared decision-making could help match delivery method to the individual’s needs and context, often using a blended approach.

*It’s about the individual and about choice… being able to have those shared decision-making conversations with someone so that you both feel that you have the choice*.

[DG2, PT1]

Despite the recognition of preference, in the survey (phase two), only 1 in 5 clinicians (10/49) said they routinely offered telerehabilitation to all service users, with most choosing to offer it selectively. This contrasts with the voice of service users (phase two), who clearly valued being involved: 100% (11/11) of those surveyed said they either wanted or had wanted to be fully involved in the decision to use telerehabilitation.

This theme highlights the importance of flexibility and personalization in the use of telerehabilitation. While clinical suitability remains critical, particularly for those with cognitive, sensory, or communication challenges, decisions are also shaped by geography, workload, and perceived confidence with technology. These findings suggest that participants did not consider telerehabilitation to be universally applicable or dismissible, and that a thoughtful approach to shared decision-making is required to meet people’s needs.

Survey items related to this theme are summarized in Multimedia Appendix 4: Survey items theme 2.

### Theme 3: Successful Use of Telerehabilitation—Support for Staff

#### Overview

This theme explores how staff can be better supported to use telerehabilitation effectively within community stroke services. While telerehabilitation was recognized as a flexible and efficient tool, its success depended on service structures that enabled collaboration, appropriate technology, and sufficient training. For some, telerehabilitation offered a welcome opportunity to work more efficiently and responsively. However, others reflected on the practical and systemic challenges they faced. As one participant put it:

*So it is an incredibly efficient way of working, but I do appreciate and we do have to do it out of necessity because we’re so pressed. But actually, I think there’s some value in working that way.*

[DG2, SLT 1]

Detailed analysis of this theme identified five subthemes: MDT communication, efficiency, technology, training, and service pressures*.*

#### MDT Communication

Participants described how telerehabilitation was used not only to engage with service users but also to collaborate with colleagues. Being able to involve other professionals in real time enhanced coordination and decision-making:

*The number of times that people write in their notes, liaise with another colleague, whereas you can do that in the here and now… We can get that piece of equipment ordered. It’s not then waiting for another visit the following week. And another lot of travel… it’s done.*

[DG2, SLT1]

This real-time input also reduced the need for repeated assessments:

*The therapist who has more speciality within that area can ask relevant questions more easily and immediately… So they’re not repeating assessments and repeating information.*

[DG2, PT1]

#### Efficiency

Improved communication contributed to greater efficiency, particularly when one staff member could be present with the service user, and another could join remotely:

*I found it quite helpful or particularly helpful if a rehab assistant was already seeing somebody and I may be needed to review a situation or review where they were with their exercise programmes that I could join, I could be in the office, but I could join that session remotely.*

[DG2, PT1]

Telerehabilitation was also seen to reduce wasted time in care home settings:

*If they’re not ready at the time of video, you can just say okay, well, call me back in 10 minutes… Whereas if you’re at the care home, you’re kind of stuck there.*

[DG3, SLT 1]

Survey results (phase two) supported these findings. Over three-quarters (37/49, 76%) of staff agreed that telerehabilitation allowed them to be more responsive in assessment. Further, 73% (36/49) of staff agreed that it enabled increased therapy intensity. Service users also valued this flexibility: 91% (10/11) of staff said they would accept telerehabilitation if it meant being seen more often or more quickly.

#### Technology

Technology was described as both an enabler and a barrier. While platforms such as WhatsApp (Meta) were familiar and easy to use, some hospital-based systems were harder to navigate:

*We've had to get really good at troubleshooting when patients… navigated the wrong way.*

[DG3, PT1]

Device type also mattered. Some participants noted that smartphones worked best for certain tasks, while laptops were preferred for others. Familiarity, accessibility, and ease of use were all seen as essential.

#### Training

Participants identified a gap in meaningful training both for themselves and for service users. While basic instructions (such as finding a quiet room) were available, more tailored and in-depth training was lacking:

*The obvious bits… were well established, but everything else… not so much.*

[DG2, SLT1]

Some staff felt that providing training to service users would improve uptake and quality. However, one person with lived experience shared a different perspective:

*From a patient’s point of view, if I’ve been told at the time that I had to find out quite space, I had to wear certain clothes, I had to do all the things that you’ve just said that …would have completely put me off full stop. I wouldn’t have done it.*

[DG2, SU1]

**Figure 3.**
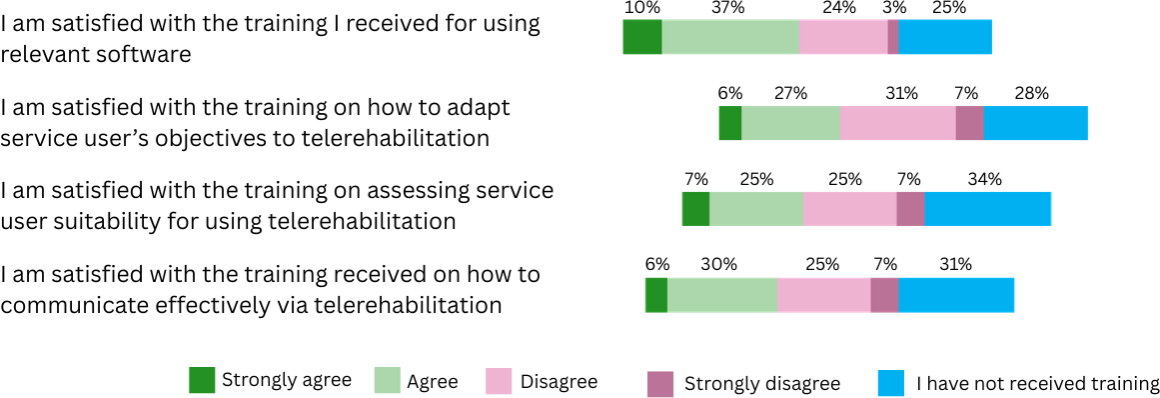
Horizontal stacked bar chart of clinical staff’s responses to four telerehabilitation training statements (software, adapting objectives, assessing suitability, and effective communication), derived from survey data from all staff delivering community stroke rehabilitation in the East of England who responded to this survey, 2023-2024 (n=67). Response categories were “strongly agree,” “agree,” “disagree,” “strongly disagree,” and “I have not received training.”

In contrast to analyses restricted to clinical staff with prior telerehabilitation experience, training-related survey items included all clinical staff in the analytical sample (n=67). Survey results (phase two) revealed varied satisfaction with current training. While 47% (35/49) of staff felt confident in their training on using relevant software, fewer were satisfied with training on adapting service-user goals (22/49, 33%), assessing suitability (22/49, 32%), or communicating effectively via telerehabilitation (24/49, 36%). Notably, staff who had never used telerehabilitation were significantly more likely to report receiving no training at all Figure 3.

#### Service Pressures

Participants reflected on how service pressures influenced the use of telerehabilitation. Some acknowledged that it was introduced or used more heavily due to workforce limitations, but still recognized its value:

*They probably get more telerehab than they would face to face. They also might get seen quicker.*

[DG3, SLT1]

In this context, telerehabilitation was not simply a compromise but a means of extending reach and offering timely support. Survey findings (phase two) supported this: 75% (37/49) of staff agreed that telerehabilitation helped them to be more responsive in assessment, and 80% (39/49) agreed it was effective for 6-month reviews.

This theme illustrates that successful telerehabilitation is closely tied to how well staff are supported to use it. Staff welcomed the increased efficiency and collaborative opportunities that telerehabilitation enabled, but identified unmet needs around training, technology, and service delivery models. With appropriate tools, shared platforms, and a needs-led approach to staff development, telerehabilitation can enhance both teamwork and service responsiveness, even in pressured environments.

Survey items supporting this theme are summarized in Multimedia Appendix 5: Survey items theme 3.

## Discussion

### Interpretation of Principal Findings

This large-scale service evaluation successfully mapped current models and experiences of telerehabilitation delivery in community stroke services by engaging relevant stakeholders, including frontline clinical staff and people with lived experience of stroke, using a sequential mixed methods approach (evaluation aim 1). It also identified practical lessons and enabling factors to support the sustained integration of telerehabilitation into routine stroke rehabilitation pathways, in line with current policy-drivers (evaluation aim 2). As a result of in-depth interrogation of data from multiple sources using recognized methods [15], we developed a conceptual model to underpin and guide the successful sustained use of telerehabilitation in community stroke services (Figure 1). The model coalesced around three themes: risks and benefits; individualized care, and support for staff.

In considering the possible risks and benefits of using telerehabilitation in community stroke services, a complex interaction between the need to reduce burden on services while still building meaningful relationships and understanding personal environments was exposed. While it was clear that immediate practical needs might be met, for clinicians, developing a sense of the person in their social environment and being effective and safe was generally viewed as more challenging using telerehabilitation than face-to-face approaches. Similarly, people with lived experience of stroke generally disagreed that telerehabilitation was comparable to face-to-face delivery. This latter point contrasts with findings of a recent systematic review of randomized-controlled trials investigating factors influencing telerehabilitation delivery in stroke [11], reporting overwhelmingly positive feedback for access to and interaction with therapists via telerehabilitation. More promisingly, in the current evaluation, on deeper interrogation within this theme, many clinicians felt able to adapt and develop rapport, considering themselves able to successfully build relationships with service users using remote communication. Additionally, some evidence of forward-planning and improved self-management by people with stroke was apparent as they prepared for telerehabilitation sessions, in line with previous work exploring possibilities for self-management through the use of technology [17]. Clinicians also recognized clear efficiency gains when telerehabilitation was used appropriately. Reduced travel time, flexible scheduling, and the ability to conduct joint sessions remotely enabled more responsive working and, in some cases, increased therapy intensity without additional service burden. For people with lived experience, albeit a small sample size here, this flexibility was viewed positively when it translated into being seen more often or more quickly. These findings suggest that although telerehabilitation can make some aspects of relationship-building more difficult, it may also offer a pragmatic way of increasing rehabilitation dose within constrained workforce capacity. Further, carefully designed research is needed to understand the nuanced characteristics that underpin this paradigm shift in delivery, in concert with the views of people with lived experience.

It is unsurprising that the need for an approach to using telerehabilitation shaped by individual needs, preferences, and circumstances was a core finding of the evaluation; that a “one-size fits all” approach was not suitable. The importance of personalized care and shared decision-making throughout patient pathways is well-established in modern health services, with improved outcomes and experiences linked to people’s involvement in shaping their care [18,19]. Here, we found that the small group of people with lived experience of stroke who wanted to be fully involved with decisions about use, and that clinicians considered multiple individual factors when deciding whether to use telerehabilitation. These not only included clinical factors such as consideration of cognitive, sensory, or severe communication challenges—often seen as “red flags” for telerehabilitation to be avoided—but decisions were also shaped by factors such as people’s personal preferences and their perceived confidence with technology. A deeper exploration of that latter point revealed similarities with previous work on health technologies in stroke and neurorehabilitation—that the challenge is not to dismiss but to support people in taking advantage of the potential benefits of technological approaches without making assumptions about age or digital literacy [20,21]. In fact, here we demonstrated that age was least important in influencing decision-making by clinical teams, but access to appropriate equipment was considered critical to digital participation and reducing inequalities of provision. This theme demonstrated that thoughtful shared decision-making, supported by access to appropriate equipment and clear communication, can help ensure that remote delivery meets the needs of diverse individuals and avoids reinforcing existing inequalities.

A prevailing body of knowledge indicates that, for both technological advancement and other wide-scale service innovation in stroke and neurorehabilitation, supportive organizational cultures are central to sustained change [21,22]; the evaluation findings presented here reflect that understanding. Specifically, clinicians identified the value of a collaborative, multidisciplinary service approach and a need for meaningful, tailored training for themselves and people with lived experience. Telerehabilitation training needs were exposed on how to work with people to adapt their goals, how to assess suitability for the approach, and how to communicate effectively online—in essence, this approach is about much more than the simple mechanics of opening a laptop or smartphone and making a call, if outcomes for both individuals and the service are to be optimal. As recently captured in an extensive scoping review [23], successful use of telerehabilitation requires a range of competencies from both people with lived experience and clinical teams. Telerehabilitation is a new model of care, implemented at pace in response to urgent need, and as such, workforce transformation through tailored development and a needs-led approach to training is required to embrace its full potential.

### Strengths and Limitations

The work met an identified need for reporting of the practicalities and challenges of use of telerehabilitation in NHS services [7] using a recognized approach to combining multiple data sources. This was a time-limited pragmatic service evaluation in a large geographical area of the United Kingdom (East of England) with community health provision across a mix of rural, suburban, and city-based populations. We acknowledge the limitation inherent in evaluating provision only within the UK NHS, but we suggest that key findings on enablers of sustained use of telerehabilitation in community stroke services have potential for application in other systems. The evaluation was supported by the existing integrated stroke delivery network, PPI advisory group, and we acknowledge that our group of participants with lived experience of stroke and their caregivers was small and therefore, while providing considerable insights, cannot represent the views of the wider service-user and caregiver populations. Despite this, we adopted an inclusive model of shared learning from service users and providers at every opportunity throughout. Where discussion groups included people with stroke, the facilitators endeavored to ensure their voices were heard. Similarly, we recognize the risk of bias inherent in surveys on a particular rehabilitation approach—that those with strong views and experiences in either direction might be most likely to respond. We made an attempt to negate this through a variety of recruitment strategies in collaboration with CD as the Stroke Quality Improvement for Rehabilitation lead. Finally, it is possible in pragmatic, rapid mixed methods evaluation that meaningful connections are lost due to limited integration or dominance of one dataset; this evaluation used recognized frameworks for study design, integration, interpretation, and reflexive approaches to minimize this risk.

### Conclusions

Digital services have clear potential to address pressing unmet rehabilitation needs as demand ever-escalates, though understanding of implementation and sustained use in a variety of service contexts has, up to now, been lacking [23]. This evaluation has made initial steps to enhance that understanding for telerehabilitation in community stroke services and should provide a platform for further primary research.

Together, the findings presented here suggest that telerehabilitation works best not as a replacement for in-person care, but as part of a responsive, blended approach grounded in individual needs and service-user choice. The evaluation demonstrates that telerehabilitation has the potential to meet people’s needs, while supporting service responsiveness and therapy intensity in pressurized clinical environments. A nuanced approach to implementation that includes appropriate training and carefully integrates the individual needs of service users and staff alongside a supportive organizational culture is required to support successful, sustained use.

## Supplementary material

10.2196/87741 Multimedia Appendix 1 Evaluation alignment with reporting guidelines for mixed methods health service research: GRAMMS (Good Reporting of a Mixed Methods Study).

10.2196/87741 Multimedia Appendix 2 Discussion groups topic guide.

10.2196/87741 Multimedia Appendix 3 Survey items theme one.

10.2196/87741 Multimedia Appendix 4 Survey items theme two.

10.2196/87741 Multimedia Appendix 5 Survey items theme three.

## Abbreviations

GRAMMS: Good Reporting of a Mixed Methods Study
MDT: multidisciplinary team
NHS: National Health Service
PPI: patient and public involvement
