# Supplementary material for: Telerehabilitation in Community Stroke Services: Mixed Methods Evaluation of Current Practice and Lessons for Sustained Use
Source: J Med Internet Res. 2026 Jun 11;28:e87741. doi: 10.2196/87741 (PMC13256497; doi:10.2196/87741)
Supplement: Multimedia Appendix 1 [file jmir-v28-e87741-s001.docx]

Evaluation alignment with reporting guidelines for mixed methods health service research: **Good Reporting of a Mixed Methods Study (GRAMMS)**

From: O’Cathain, A., Murphy, E., Nicholl, J. The quality of mixed methods studies in health services research; *Journal of Health Services Research and Policy* 2008*:* 13(2): 92-98 [10.1258/jhsrp.2007.007074](https://doi.org/10.1258/jhsrp.2007.007074)

| Reporting guidance | Alignment with manuscript |
| --- | --- |
| 1. Describe the justification for using a mixed methods approach to the research question | The justification for a mixed-methods approach, specifically an exploratory sequential mixed-methods evaluation, is articulated in the opening paragraph of the study design section and supported with appropriate citation, within the study methods. |
| 1. Describe the design in terms of the purpose, priority, and sequence of methods | The exploratory sequential design is described in the opening paragraph of the study design section, within the study methods. Then, the purpose and sequence of methods are further described in the final paragraph of the study design section within the methods. The connections between phase one of the work (qualitative), and how it informed the conceptual framework and survey design for phase two, are similarly presented in the study design section. |
| 1. Describe each method in terms of sampling, data collection and analysis | Details of sampling, data collection and analysis are articulated in the methods section of the paper, with detail for each phase of the work given in the recruitment, data collection and analysis sections. |
| 1. Describe where integration has occurred, how it has occurred and who has participated in it | According to the exploratory sequential design adopted, qualitative methods preceded quantitative; and integration occurred during analysis, interpretation and reporting. Details of integration are reported in the final paragraph of the study design section of the methods. Details of who participated in each stage are presented in the data collection section of the methods, and in the opening section of the results. The use of integration through narrative is articulated in the opening to the evaluation findings section of the results. Evaluators involved in thematic analysis and interpretation of the survey are initialled in the methods section where appropriate. |
| 1. Describe any limitation of one method associated with the present of the other method | This is included in the limitations section, and mitigations to risks of insufficient and imbalanced integration of the focus groups and survey findings are evident in the descriptions of the study design and presentation of evaluation findings. |
| 1. Describe any insights gained from mixing or integrating methods. | Using integration through narrative, multiple insights are captured throughout the presentation of evaluation findings section. Those insights are expanded upon throughout the discussion, in alignment with evaluation aims. |
