# Supplementary material for: Telerehabilitation in Community Stroke Services: Mixed Methods Evaluation of Current Practice and Lessons for Sustained Use
Source: J Med Internet Res. 2026 Jun 11;28:e87741. doi: 10.2196/87741 (PMC13256497; doi:10.2196/87741)
Supplement: Multimedia Appendix 2 [file jmir-v28-e87741-s002.docx]

Part 1:

Welcome and Introduction (5mins)

- Introduce discussion group facilitators.
- Confirm the discussion will be audio recorded.
- Explain the purpose of the group.
- Explain the ground rules:
  - Reiterate no names will be associated with anything said and no one will have access to the recording apart from the evaluation team.
  - As that participants participate actively in the discussion,
  - speak one at a time,
  - treat everyone’s ideas with respect,
  - avoid side conversations,
  - keep focused on the topic.
- Invite participants to help themselves to refreshments.

Part 2 (55mins)

| Question Type | Purpose | Question |
| --- | --- | --- |
| Introduction | To begin discussion | What was your experience of community stroke rehabilitation during Covid lockdowns? |
|  | Prompt for discussion | Did you visit your patients/ were you visited as normal? |
|  |  | What has changed since the pandemic? |
| Theme 1 | Explore what is telerehabilitation | What does telerehabilitation mean to you? |
|  | Prompt for discussion | What are your experiences of telerehabilitation? |
|  |  |  |
| Theme 2 | Explore when is telerehabilitation best used | Can you tell me about an occasion when you used or received telerehabilitation? |
|  | Prompt for discussion | What went well? |
|  |  | What could have been better? |
|  |  | Do you think telerehabilitation works better for some people than others? Why? Who? |
|  |  | How do you (clinicians) decide when to use telerehabilitation? |
|  |  | When is it not suitable? |
|  |  | When would you (patients) like to receive telerehabilitation and when would you prefer a face-to-face visit? |
|  |  |  |
| Theme 3 | Explore the technology available for telerehabilitation | What technology options are there for delivering telerehabilitation? |
|  | Prompt for discussion | Is the technology easy to use for stroke survivors, carers and clinicians? |
|  |  | What would you say are the strengths and negatives of different technologies in terms of the delivery of community stroke rehabilitation? |
|  |  | How do you arrange a telerehabilitation visit? |
|  |  | Does the technology link to NHS systems? |
|  |  |  |
| Theme 4 | Explore the value of telerehabilitation | Is community stroke rehabilitation as effective when delivered by telerehabilitation as it would be face-to-face? |
|  | Prompt for discussion | How does the use of telerehabilitation (either delivering or receiving) rather than a face-to-face visit impact on your day? |
|  |  | Does using telerehabilitation change the relationship between patient and NHS team? |
|  |  | Is there any impact on contact time between the patient and NHS team? |
|  |  |  |
| Closing points | Capture remaining ideas | Thinking back on everything we have discussed today what could be done to make telerehabilitation better? |
