# Supplementary material for: Telerehabilitation in Community Stroke Services: Mixed Methods Evaluation of Current Practice and Lessons for Sustained Use
Source: J Med Internet Res. 2026 Jun 11;28:e87741. doi: 10.2196/87741 (PMC13256497; doi:10.2196/87741)
Supplement: Multimedia Appendix 3 [file jmir-v28-e87741-s003.docx]

**Survey Items Informing Theme 1 – Successful Use of Telerehabilitation: Risks and Benefits**

| **Subtheme** | **Survey Item** | **Response Summary** |
| --- | --- | --- |
| **The Environment** | *Initial assessments should always be face-to-face* | 22% (n=11) Agree Strongly, 31% (n=15) Agree, 37% (n=18)Disagree, 10% (n=5) Disagree Strongly |
|  | *Telerehabilitation can be as safe as face-to-face rehabilitation* | 2% (n=1) Agree Strongly, 43% (n=21) Agree, 41% (n=20) Disagree, 14% (n=7) Disagree Strongly |
|  | *Telerehabilitation can be as effective as face-to-face therapy* | 4% (n=2) Agree Strongly, 33% (n=16) Agree, 41% (n=22)Disagree, 18% (n=9)Disagree Strongly |
| **Relationships** | *I can build rapport using telerehabilitation* | 6% (n=3) Agree Strongly, 63% (n=31) Agree, 27% (n=13) Disagree, 4% (n=2) Disagree Strongly |
|  | *Telerehabilitation enables me to share decision making with service users* | 10% (n=5) Agree Strongly, 76% (n=37) Agree, 8% (n=4) Disagree, 6% (n=3) Disagree Strongly |
| **Working Holistically** | *Telerehabilitation helps me to support the long-term needs of service-users* | 10% (n=5) Agree Strongly, 63% (n=31) Agree, 20% (n=10) Disagree, 6% (n=3) Disagree Strongly |
|  | *Telerehabilitation encourages service-users to take ownership of their rehabilitation* | 8% (n=4) Agree Strongly, 55% (n=27) Agree, 27% (n=13) Disagree, 10% (n=5) Disagree Strongly |
| **Service-user view** | *Telerehabilitation was/is of equal quality to face-to-face therapy* (Stroke survivors and carers) | 0% (n=0) Agree Strongly, 36% (n=4) Agree, 45% (n=5) Disagree, 18% (n=2) Disagree Strongly |

Note: Percentages may not total exactly 100% due to rounding. Participants could select levels of agreement using a four-point Likert scale. The final row reflects responses from stroke survivors (n=10) and one unpaid carer (n=1), who were asked a slightly adapted version of the question used for staff.
