# Supplementary material for: Telerehabilitation in Community Stroke Services: Mixed Methods Evaluation of Current Practice and Lessons for Sustained Use
Source: J Med Internet Res. 2026 Jun 11;28:e87741. doi: 10.2196/87741 (PMC13256497; doi:10.2196/87741)
Supplement: Multimedia Appendix 4 [file jmir-v28-e87741-s004.docx]

**Survey Items Informing Theme 2 – Individualised Care**

| **Subtheme** | **Survey Item** | **Response Summary** |
| --- | --- | --- |
| **Selection and triage** | *How has your use of telerehabilitation changed since the end of Covid restrictions?* | 57% (n=28) use it when necessary; 12% (n=6)stopped completely; 20% (n=10) use it to connect with colleagues |
|  | *I know which service users will benefit most from telerehabilitation* | 14% (n=7) Agree Strongly; 71% (n=35) Agree; 12% (n=6) Disagree; 2% (n=1) Disagree Strongly |
|  | *I offer the option of telerehabilitation to those service users that I think might like it* | 37%(n=18) selected |
|  | *I still routinely offer the option of telerehabilitation to service users on my caseload* | 20% (n=10)selected |
|  | *Ranking question: Which factors are most important in determining suitability for telerehabilitation?* | Top-ranked: access to IT, service user preference, ability to engage online |
| **Perceptions and expectations** | *Service users consider telerehabilitation of equal quality to face-to-face therapy* | 0% (n=0) Agree Strongly; 14% (n=7) Agree; 76%(n=37) Disagree; 10% (n=5)Strongly Disagree |
|  | *Service users enjoy participating in telerehabilitation* | 2% (n=1) Agree Strongly, 39% (n=19) Agree; 55% (n=27) Disagree; 4% (n=2) Disagree Strongly |
|  | *Service users are pleased to be offered telerehabilitation as a service option* | 2% (n=1) Strongly Agree; 65% (n= 32) Agree; 31% (n= 15) Disagree; 2% (n=1) Disagree Strongly. |
| **Inequalities** | *Ranking question: Access to IT equipment* | Ranked as most important factor in determining suitability |
|  | *I am confident with technology* | 31% (n=15) Agree Strongly; 55% (n= 27) Agree; 14% (n= 7) Disagree; 0% (n=0) Disagree Strongly |
| **Preferences Service-user view** | *I was/would want to be fully involved in the decision to have telerehabilitation (service users/carers only)* | 36% (n=4) Strongly Agree; 63% (n=7) Agree; 0% (n=0) Disagree’ 0% (n=0) Strongly Disagree |
|  | *I don’t mind the principle of telerehabilitation (service users/carers only)* | 36% (n=4) Agree Strongly, 36% (n=4) Agree; 18% (n=2) Disagree; 9% (n=1) Disagree Strongly |
|  | *I was pleased/would have been pleased to be offered telerehabilitation (service users/carers only)* | 36% (n=4) Strongly Agree; 27% (n=3) Agree; 27% (n=3) Disagree; 9% (n=1) Disagree Strongly |
|  | *It has become part of normal life to be offered digital appointments (service users/carers only)* | 18% (n=2) Agree Strongly; 55% (n=6) Agree; 18% (n=2) Disagree; 9% n=1) Disagree Strongly |
|  | *I am confident with technology* | 45% (n=5) Agree Strongly; 27% (n=3) Agree; 18% (n=2) Disagree; 9% (n=1) Disagree Strongly |
|  | *I am/would have been comfortable using telerehabilitation* | 36% (n=4) Agree Strongly; 45% (n=5) Agree;9% (n=1) Disagree; 9% (n=1) Disagree Strongly |

Note: Some items were filtered by respondent type (e.g. staff only, or stroke survivors and unpaid carers only). Percentages may not total 100% due to rounding or multiple selections.
