# Supplementary material for: Telerehabilitation in Community Stroke Services: Mixed Methods Evaluation of Current Practice and Lessons for Sustained Use
Source: J Med Internet Res. 2026 Jun 11;28:e87741. doi: 10.2196/87741 (PMC13256497; doi:10.2196/87741)
Supplement: Multimedia Appendix 5 [file jmir-v28-e87741-s005.docx]

**Survey Items Informing Theme 3 – Support for Staff**

| **Subtheme** | **Survey Item** | **Response Summary** |
| --- | --- | --- |
| Efficiency | Telerehabilitation enables us to increase intensity (n=49) | 12% (n=6) Agree Strongly; 61% (n=30) Agree; 20% (n=10) Disagree; 6% (n=3) Disagree Strongly |
|  | Telerehabilitation allows us to be responsive in assessment (n=49) | 20% (n=10) Agree Strongly; 55% (n=27) Agree; 18% (n=9) Disagree; 6% (n=3) Disagree Strongly |
|  | Telerehabilitation is effective for 6-month reviews (n=49) | 10% (n=5) Agree Strongly; 69% (n=34) Agree; 12% (n=6)Disagree; 8% (n=4)Disagree Strongly |
| Service-user views | I would accept telerehabilitation if I/my family member was seen more often (n = 11) | 46% (n=5) Agree Strongly; 46% (n=5) Agree; 0% (n=0) Disagree; 9% (n=1) Disagree Strongly |
|  | I would accept telerehabilitation if I/my family member could be seen quicker (n = 11) | 46% (n=5) Agree Strongly; 46% (n=5) Agree; 0% (n=0) Disagree; 9% (n=1) Disagree Strongly |
| Training | I am satisfied with the training for using relevant software (n = 67) | 10% (n=7) Agree Strongly; 37% (n=25) Agree; 24% (n=16) Disagree; 3% (n=2) Disagree Strongly; 25% (n=17) No Training |
|  | I am satisfied with training on adapting service-user objectives to telerehabilitation (n = 67) | 6% (n=4) Agree Strongly; 27% (n=18) Agree; 31% (n=21) Disagree; 7% (n=5) Disagree Strongly; 28% (n=19) No Training |
|  | I am satisfied with training on assessing service-user suitability (n = 67) | 7% (n=5) Agree Strongly; 25% (n=17) Agree; 25% (n=17) Disagree; 7% (n=5) Disagree Strongly; 34% (n=23) No Training |
|  | I am satisfied with training on communicating effectively via telerehabilitation (n = 67) | 6% (n=4) Agree Strongly; 30% (n=20) Agree; 25% (n=17) Disagree; 7% (n=5) Disagree Strongly; 31% (n=21)No Training |
| Service pressures | Telerehabilitation enables us to provide a 7-day a week service (n=49) | 6% (n=3) Agree Strongly; 20% (n=10) Agree; 57% (n=28) Disagree; 16% (n=8) Disagree Strongly |
|  | Increased use of telerehabilitation would support a 7-day a week service (n=49) | 6% (n=3) Agree Strongly; 43% (n=21) Agree; 37% (n=18) Disagree; 14% (n=7) Disagree Strongly |

Note: Percentages may not total exactly 100% due to rounding. Most items were filtered to include only staff who had used telerehabilitation (n = 49), except for training items which were shown to all staff (n = 67). Service-user responses reflect only those with experience of telerehabilitation (n = 11).
